# Advances in ribosome profiling technologies

**DOI:** 10.1042/BST20253061

**Published:** 2025-05-16

**Authors:** Kotaro Tomuro, Shintaro Iwasaki

**Affiliations:** 1 RNA Systems Biochemistry Laboratory, Pioneering Research Institute, RIKEN, Wako, Saitama 351-0198, Japan; 2 Department of Computational Biology and Medical Sciences, Graduate School of Frontier Sciences, The University of Tokyo, Kashiwa, Chiba 277-8561, Japan

**Keywords:** deep sequencing, ribo-seq, ribosome profiling, translational control

## Abstract

Ribosome profiling (or Ribo-seq) has emerged as a powerful approach for revealing the regulatory mechanisms of protein synthesis, on the basis of deep sequencing of ribosome footprints. Recent innovations in Ribo-seq technologies have significantly enhanced their sensitivity, specificity, and resolution. In this review, we outline emerging Ribo-seq derivatives that overcome barriers in low inputs, rRNA contamination, data calibration, and single-cell applications. These advances enable detailed insights into translational control across diverse biological contexts.

## Introduction

**Figure 1:**
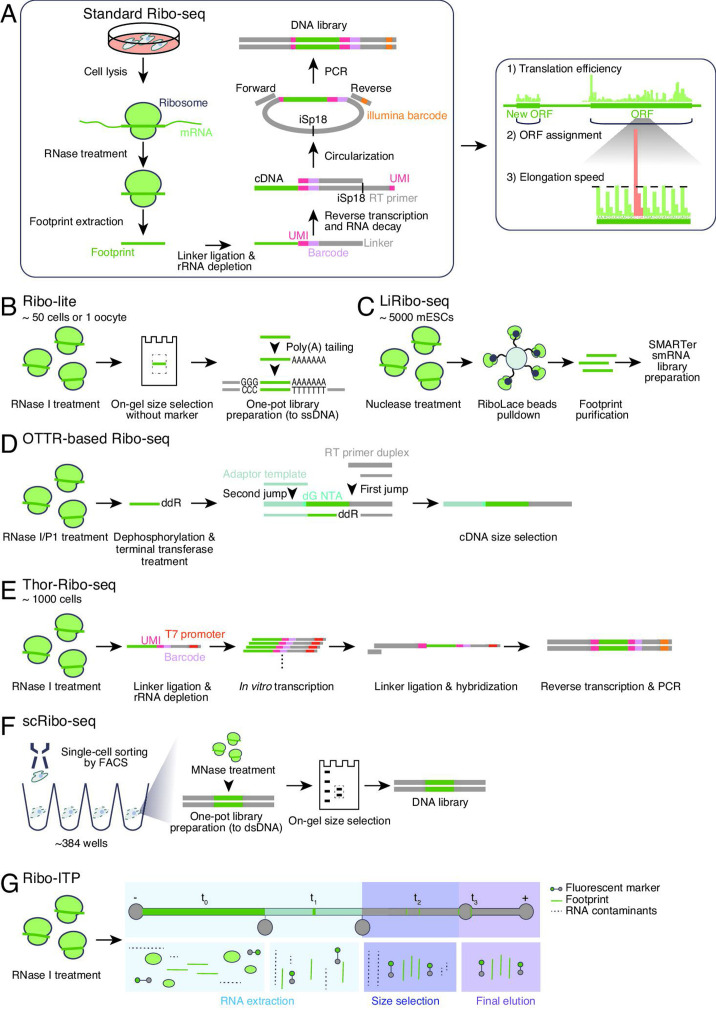
Ribo-seq derivatives for ultralow inputs and single individual cells. (**A**) Schematic of library preparation strategy for conventional Ribo-seq (left) and the assessment of translational status (right). (**B–E**) Schematic of library preparation strategy for methods tailored for ultralow inputs. (**F–G**) Schematic of library preparation strategy for single-cell Ribo-seq. iSp18, a hexa-ethylene glycol spacer; ddR, 2′, 3′-dideoxyadenosine or 2′, 3′-dideoxyguanosine; dG, deoxyguanosine; dsDNA, double-stranded DNA, FACS, fluorescence-activated cell sorting; NTA, nontemplated nucleotide addition; RT, reverse transcription; ssDNA, single-stranded DNA; UMI, unique molecular identifier.

The translation of mRNAs is a fundamental step in gene expression, which orchestrates the production of functional proteins. Translation is increasingly recognized as a dynamic layer of gene regulation that influences how cells respond to development [1,2], stress [3], and disease [4,5]. Since its development, ribosome profiling (or Ribo-seq), a technique based on deep sequencing of ribosome-protected mRNA fragments generated by RNase treatment [6], has opened new avenues for measuring translation across the transcriptome, or the ‘translatome’, in various biological contexts [7–10] (Figure 1A). This technique reveals translational efficiency, identifies new open reading frames (ORFs), and monitors ribosome traversal speed at codon resolution in a genome-wide manner (Figure 1A). Even after 15 years of research, translatomic studies have shown no signs of ending. However, the inherent technical limitations of Ribo-seq that limit its applications have become evident. This review summarizes the drawbacks and recent technological advancements designed to address these issues.

### Expanding Ribo-seq to ultralow-input samples

One of the clear challenges in Ribo-seq is applying it to low inputs. Conventional Ribo-seq protocols require ~10^6^ or more cells [11,12] and have difficulties utilizing a small number of cells as starting materials.

Given that step-by-step sample loss is one of the major obstacles for low inputs, a one-pot reaction is an effective option. For this purpose, ligation-free methods have been implemented [13–17]. Typically, ribosome footprints are tailed with A nucleotides by poly(A) polymerase and reverse-transcribed with oligo deoxythymidine (dT) primers. During reverse transcription (RT), another linker sequence is conjugated through a template-switching mechanism. Among these studies, ligation-free, ultralow-input, and enhanced Ribo-seq (termed Ribo-lite) is further optimized by the appropriate concentration of RNase I—a widely used RNase for Ribo-seq, the skipping of rRNA depletion to suppress sample loss, and on-gel size selection without size markers that often contaminate libraries [15] (Figure 1B). Ribo-lite was applied to low-inputs, such as 1,000 HEK293 cells and 100 mouse oocytes, and even ultralow-inputs, such as 50 HEK293 cells and a single oocyte. The combination of Ribo-lite with Smart-seq2 (a low-input RNA-seq protocol) [18] allows simultaneous analysis of the translatome and transcriptome in human oocytes and embryos [termed Ribo-RNA-lite (R2-lite)] [16].

A low-input ribosome profiling (LiRibo-seq) technique [17] also employs ligation-free one-pot library preparation but has a unique method of footprint recovery after RNase digestion. With biotin-conjugated puromycin, which is covalently linked to a nascent peptide chain through a peptidyl transferase reaction by the ribosome, footprint-ribosome-biotinylated puromycin complexes are isolated by streptavidin beads (the method termed RiboLace) [19] (Figure 1C). LiRibo-seq measures the translatome in 5,000 mouse embryonic stem cells and the maternal-to-zygotic transition in mouse embryos [17].

Similarly, serial template-switching reactions during RT allow one-pot reactions. In the ordered two-template relay (OTTR) [20,21] (Figure 1D), ribosome footprints are treated with terminal transferase to add a dideoxynucleotide at the 3′ end and used for template jumping cDNA synthesis by modified reverse transcriptase from eukaryotic retroelements and the first linker. Subsequently, second template switching is induced with the second linker. The difference in hybridizing nucleotide species in the first (3′ A/G at the footprint template with 3′ T/C for the first primer) and second (3′ C for the DNA template with 3′G for the cDNA extension) template-switching reactions avoids concatamerization [20]. This technique is also helpful in reducing the amount of material required for Ribo-seq with limited bias [21].

Another strategy to address limited materials is to amplify nucleic acids at the early step of library preparation. The T7 high-resolution original RNA (Thor) technique [22] (originally described by Lexogen) employs RNA-dependent RNA amplification by T7 RNA polymerase, using an RNA‒DNA chimera as an *in vitro* transcription template (Figure 1E). Owing to the linear increase in RNA at the beginning rather than before the final PCR step, Thor-Ribo-seq can minimize the risk of material loss during library preparation. Strikingly, Thor-Ribo-seq maintains high data reproducibility for a wide range of inputs from ~10^6^ to ~10^3^ cultured cells [22]. A successful application has been reported in dissected fly testes [23].

A potential technical limitation of ultralow-input Ribo-Seq may lie in the annotation of novel ORFs. Although Ribo-seq has been used to define ORFs *de novo*, this analysis generally requires high read coverage along ORFs. Given the restricted RNA molecule complexity in the low-input materials, Ribo-seq data from such samples may offer limited footprint coverage, even with high sequencing depth. Thus, some difficulty in ORF annotation from low-input data could be expected.

### Measuring the single-cell translatome

Since conventional Ribo-seq is prepared from the lysate of bulk cells, the profiles should present the average translatome for many cells that may intrinsically have variations in translation. Two independent Ribo-seq methods tailored for single-cell-level measurement have been developed [24,25].

The van Oudenaarden group developed single-cell ribosome sequencing (scRibo-seq) [24] (Figure 1F). In this method, individual cells are collected in a 384-well plate by a cell sorter. The single-pot experiment involves cell lysis, RNase digestion by micrococcal nuclease (MNase), two consecutive linker ligations to the 3′ end and the 5′ end of RNA fragments, RT, and PCR amplification with sample barcode addition. All the samples in the plate are pooled, gel-purified, and sequenced. Footprint differences across ORFs among cells are analyzed by well-established tools for single-cell RNA-seq, such as Seurat [26]. The advantage of MNase, which degrades both DNA and RNA, is the stringent control of activity by Ca^2+^ ions; this chelation of the ion completely quenches the activity—otherwise, linker DNAs or cDNAs would also be digested by the enzyme. The disadvantage of MNase is an A/U preference for cleavage [27], hampering the assessment of the boundary of ribosome coverage from the reads. Understanding the edge of ribosome footprints is extremely important for the estimation of the A-site position in the ribosome and thus for codon-wise evaluation of ribosome traversal. To overcome this issue, a random forest classifier is trained to correct for the sequence bias introduced by MNase digestion and is used to assign the A-site location in the reads [24].

Following the development of scRibo-seq, the Cenik group developed a microfluidic isotachophoresis (ITP)-based technique, termed Ribo-ITP [25] (Figure 1G). This system uses a high-yield microfluidic system for RNA purification and footprint enrichment; RNase-treated cell lysate is subjected to ITP together with fluorophore-conjugated marker oligonucleotides, and then, ribosome footprints of the corresponding size are selected. Starting with a sorted single cell, enriched footprints are handled with the ligation-free method [poly(A)-tailing and reverse-transcription with template switching similar to Ribo-lite]. Importantly, Ribo-ITP substantially reduces the sample processing time and the amount of sample needed. Using Ribo-ITP, the authors characterized allele-specific translation of zygotic transcripts during early mouse embryogenesis [25].

Technically, those single-cell techniques have a chance to employ techniques tailored for ultralow-inputs, such as OTTR [21] and Thor [22]. Notably, since both scRibo-seq and Ribo-ITP methods (and Ribo-lite) skip the rRNA depletion step (see below for details), restricted read depth may be one drawback.

### Breakaway from relative analysis

Another challenge in Ribo-seq is the quantification of global translation changes. Owing to the nature of deep sequencing-based measurement, the evaluation is always ‘relative’; if translational changes are restricted to a subset of mRNA, the standard relative enrichment/depletion analysis is adequate. However, overall protein synthesis can often be substantially altered; for example, global translation shutoff is associated with stress [28,29] and translation inhibitor treatment [30,31], coupled with mRNA-selective effects. Generally, the accurate track of the global shift in Ribo-seq or, more broadly deep sequencing-based methods, is challenging [32–34].

A straightforward strategy to address this situation is the addition of ‘spike-in’ control. In RNA-seq, this process is relatively easy [35] since the artificial RNAs for this purpose are commercially available (such as the External RNA Controls Consortium spike-in control). However, for Ribo-seq, the preparation of such external standards is difficult.

**Figure 2:**
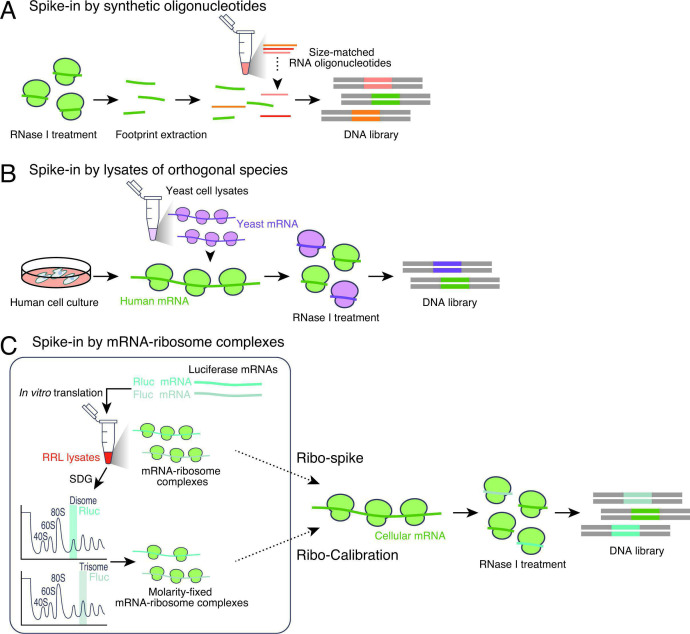
Spike-in controls to monitor global translation changes. (**A-C**) Schematic of strategy for spike-in controls used in Ribo-seq experiments. Synthesized short RNA oligonucleotides could be added after ribosome footprint recovery (**A**). Alternatively, lysate of orthogonal species (**B**) or ribosome-mRNA complexes generated by *in vitro* translation (**C**) could be added to the cell lysate before RNase digestion. Fluc, firefly luciferase; Rluc, *Renilla* luciferase; RRL, rabbit reticulocyte lysate; SDG, sucrose density gradient.

Several approaches have been developed for spike-ins in Ribo-seq. One example is the addition of molar amount-defined, short synthetic RNA oligonucleotides to samples after RNase digestion [36–40] (Figure 2A). This oligonucleotide-based method should be performed with caution, as it assumes no variance in processes before spike-in addition or during RNase digestion and ribosome footprint recovery. For rigorous evaluations, sequence diversity or the number of RNA species should also be carefully considered.

The alternative method is based on the supplementation of lysates of orthogonal species as spike-in (e.g., yeast lysate spike-in for human Ribo-seq) [41–45] (Figure 2B). Since experiments start with the lysate mixture before RNase digestion and downstream, there is no risk for sample-to-sample variations, as with short RNA spike-ins. Moreover, the high sequence diversity in footprints from orthogonal species is advantageous for suppressing potential sequencing bias for a small subset of RNAs.

Similarly, footprints stemming from mitochondrial ribosomes, which are typically included in lysates in addition to cytosolic ribosomes, could be used for internal spike-ins [14,30,46–49]. Notably, this method is only applied when it is reasonable to assume that translation within the organelle is unaffected under the experimental conditions.

Another spike-in option harnesses purified mRNA‒ribosome complexes. This method, termed Ribo-Calibration [50], uses artificial mRNAs (such as those encoding *Renilla* luciferase or firefly luciferase) complexed with an explicit number of ribosomes (Figure 2C). After *in vitro* translation with a rabbit reticulocyte lysate system, the reaction is subjected to a sucrose density gradient to purify complexes with a defined stoichiometric ratio of ribosome-mRNA (e.g., two ribosomes/disomes or three ribosomes/trisomes). These mRNA‒ribosome complexes can be added to the lysate before RNase digestion to minimize unintended variation. With Ribo-Calibration, absolute changes in translation have been measured in various cell types or stress conditions, such as heat shock and aging [50]. Ribo-spike, which is essentially based on the same rationale [51] (Figure 2C), also allows the assessment of global translation changes during the integrated stress response.

In addition to assuming a global change in translation, Ribo-Calibration measures the mean ribosome number loaded across mRNAs; through combination of RNA-seq data from the same samples, the absolute ribosome density on mRNAs is measured, referring to the ribosome numbers on the spike-in mRNAs as 2 (disome):3 (trisome) [50]. Moreover, given the translational elongation rate measured by ribosome run-off assay-coupled Ribo-seq [52], ribosome numbers on ORFs could be converted into translation initiation rates [50]. The stoichiometry and kinetics in HEK293 cells fall into~5 ribosomes every ~270 nt; ~22 sec/event translation initiation; and ~4.1 codon/sec translation elongation as a global average, which are consistent with the values obtained by in-cell nascent chain tracking in real time [53]. Furthermore, given the mRNA half-lives that can be measured by other sequencing-based methods, such as 5′-bromo-uridine immunoprecipitation chase–deep sequencing (BRIC-seq) [54,55], the initiation rates lead to the calculation of lifetime translation rounds of mRNAs (~1,800 times translation before mRNA decay on average).

### Strategies to reduce rRNA contamination

**Figure 3:**
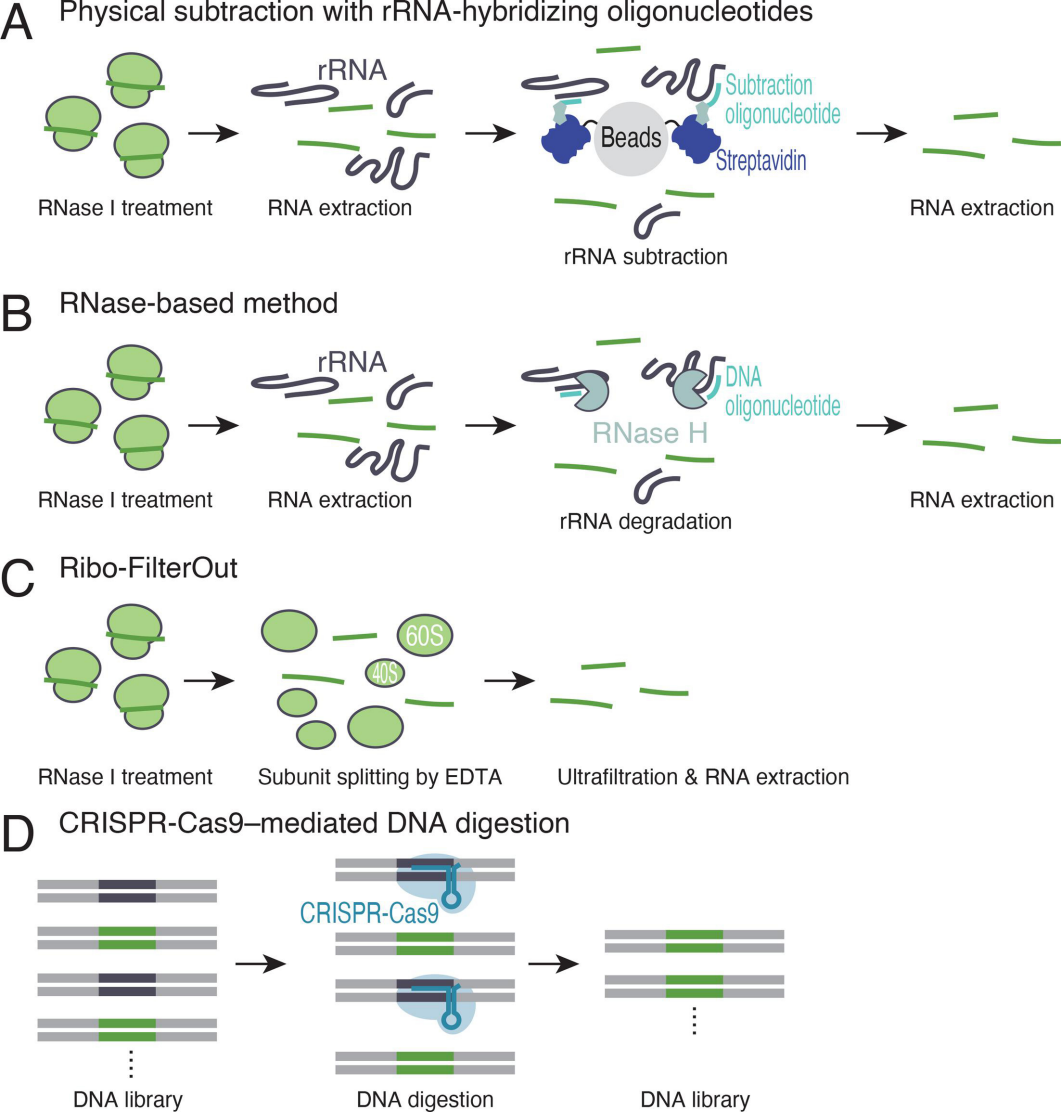
rRNA removal methods in Ribo-seq. (**A-D**) Schematic of strategy for rRNA depletion methods. rRNA are physically subtracted on streptavidin beads conjugated with hybridizing oligonucleotides (**A**) or digested by RNaseH with DNA oligonucleotide (**B**) after RNA extraction. Alternatively, rRNAs complexed in ribosome subunits are separated from ribosome footprints by EDTA treatment and ultrafiltration after RNase digestion (**C**). DNA library could be treated with Clustered Regularly Interspaced Short Palindromic Repeats (CRISPR)-Cas9 complexes with designed guide RNA targeting to rRNA fragments (**D**).

Technically, one disadvantage of Ribo-seq is the limited sequencing space due to the extensive contamination of rRNAs. For the reduction in rRNA in libraries, the conventional approach is physical subtraction with rRNA-hybridizing oligonucleotides and subsequent immobilization on streptavidin-conjugated beads [11,12,56–59] (Figure 3A) (typically 15–20% usable reads in mammalian tissue culture). Alternatively, RNase-based methods for digesting selected rRNA fragments can be used [60–62] (10–30% usable reads in mammalian tissue culture, depending on the commercial kits or methods) (Figure 3B), although the rRNA pulldown-and-subtraction method is generally suitable for Ribo-seq [58].

While these strategies focus on purified RNA fragments, a recently reported method allows rRNA reduction at an earlier step with an orthogonal rationale. After ribosome‒footprint complex isolation by ultracentrifugation, Ribo-FilterOut [50] splits it into subunits and the footprint by EDTA and then separates the footprint from the subunits through subsequent ultrafiltration (Figure 3C). This process effectively removes fragmented rRNA maintained within subunits. Importantly, the total time required to complete these steps is typically less than 30 minutes, and no expensive kits are needed. The efficacy of Ribo-FilterOut has been demonstrated by a ~16-fold increase in usable reads in combination with commercially available rRNA pulldown-and-subtraction kits (e.g., Illumina's Ribo-Zero and siTOOLs Biotech’s riboPOOL) [50] (80% usable reads in human tissue culture).

Another method focuses on the double-stranded DNA (dsDNA) library after PCR amplification instead of RNAs. Through the use of recombinant Cas9 protein and specifically designed guide RNA to target contaminated rRNA fragments, the undesired portion of dsDNA derived from rRNA is selectively cleaved from the library [12,63–65] (30–35% usable reads in human tissue culture and mouse brain) (Figure 3D).

### Probing unique ribosome configurations

RNase selection is an important factor in defining the quality of the data. *Escherichia coli* RNase I is the most common RNase for Ribo-seq due to low nucleotide bias. However, this enzyme nicks rRNAs within ribosomes and affects the integrity of the complex [66]. Ingolia’s group reported that RNase P1, which generally lacks nucleotide specificity for cleavage [67], preserves the integrity of ribosomes better than does RNase I [21] (Figure 1D); this enzyme minimizes the cleavage of rRNAs and strongly reduces rRNA contamination in the final libraries. Thus, RNase P1–Ribo-seq monitors the subpopulation of the ribosomal complex that conventional RNase I–Ribo-seq misses—a class of footprints longer than monosomes but shorter than colliding 80S–80S complexes or disomes [21]. The ‘subdisome’ footprint may represent 80S‒40S complexes or 80S complexes with accessory proteins.

### Exploring new applications of Ribo-seq

As summarized in this review, recent technical advances are overcoming the limitations of Ribo-seq and expanding its applications to a wide variety of samples with low inputs, single cells, and valuable materials. Measurements of global translation changes, kinetic assessments, and probing of ribosome configurations with increased sequencing space have improved our understanding of protein synthesis. These techniques could be combined with Ribo-seq derivatives for ribosome subsets associated with specific factors [68], scanning preinitiation complexes [69], ribosome collision [12], organelle translation in mitochondria [70] and chloroplasts [71], nascent mRNAs [72], and microbiomes [73].

**Perspectives**

- Since the development of Ribo-seq, a technique based on deep sequencing of ribosome footprints generated by RNase treatment, our understanding of the cellular translatome is expanding in unprecedented ways. Recent technical improvements overcame the obstacles that have impeded applications of Ribo-seq.
- Single-cell Ribo-seq can address the heterogeneity of cellular translation at single-cell resolution. Additionally, new protocols addressing inherent problems associated with Ribo-seq, such as high material requirements, rRNA contamination, and a lack of global quantification methods, have strengthened its power and applicability in many biological fields.
- The newly developed Ribo-seq derivatives may help our understanding of translational dysregulations associated with diseases. The further Ribo-seq application to massive parallel samples, zooming down to the subcellular or organelle level, and even obtaining single-mRNA resolution will further boost the development of therapeutic tools.

## Abbreviations

ITP: isotachophoresis
MNase: micrococcal nuclease
OTTR: ordered two-template relay
RT: reverse transcription
dsDNA: double-stranded DNA
scRibo-seq: single-cell ribosome sequencing

## References

[1] Yang G., Xin Q., Dean J (2024). Degradation and translation of maternal mRNA for embryogenesis. Trends Genet..

[2] Mercer M., Jang S., Ni C., Buszczak M (2021). The dynamic regulation of mRNA translation and ribosome biogenesis during germ cell development and reproductive aging. Front. Cell Dev. Biol..

[3] Advani V.M., Ivanov P (2019). Translational control under stress: reshaping the translatome. Bioessays.

[4] Kapur M., Ackerman S.L (2018). mRNA translation gone awry: translation fidelity and neurological disease. Trends Genet..

[5] Fabbri L., Chakraborty A., Robert C., Vagner S (2021). The plasticity of mRNA translation during cancer progression and therapy resistance. Nat. Rev. Cancer.

[6] Ingolia N.T., Ghaemmaghami S., Newman J.R.S., Weissman J.S (2009). Genome-wide analysis in vivo of translation with nucleotide resolution using ribosome profiling. Science.

[7] Brar G.A., Weissman J.S (2015). Ribosome profiling reveals the what, when, where and how of protein synthesis. Nat. Rev. Mol. Cell Biol..

[8] Iwasaki S., Ingolia N.T (2017). The growing toolbox for protein synthesis studies. Trends Biochem. Sci..

[9] Ingolia N.T., Hussmann J.A., Weissman J.S (2019). Ribosome profiling: global views of translation. Cold Spring Harb. Perspect. Biol..

[10] Wang Y., Zhang H., Lu J (2020). Recent advances in ribosome profiling for deciphering translational regulation. Methods.

[11] McGlincy N.J., Ingolia N.T (2017). Transcriptome-wide measurement of translation by ribosome profiling. Methods.

[12] Mito M., Mishima Y., Iwasaki S (2020). Protocol for disome profiling to survey ribosome collision in humans and zebrafish. STAR Protoc..

[13] Hornstein N., Torres D., Das Sharma S., Tang G., Canoll P., Sims P.A (2016). Ligation-free ribosome profiling of cell type-specific translation in the brain. Genome Biol..

[14] Li Q., Yang H., Stroup E.K., Wang H., Ji Z (2022). Low-input RNase footprinting for simultaneous quantification of cytosolic and mitochondrial translation. Genome Res..

[15] Xiong Z., Xu K., Lin Z., Kong F., Wang Q., Quan Y. (2022). Ultrasensitive Ribo-seq reveals translational landscapes during mammalian oocyte-to-embryo transition and pre-implantation development. Nat. Cell Biol..

[16] Zou Z., Zhang C., Wang Q., Hou Z., Xiong Z., Kong F. (2022). Translatome and transcriptome co-profiling reveals a role of TPRXs in human zygotic genome activation. Science.

[17] Zhang C., Wang M., Li Y., Zhang Y (2022). Profiling and functional characterization of maternal mRNA translation during mouse maternal-to-zygotic transition. Sci. Adv..

[18] Picelli S., Faridani O.R., Björklund A.K., Winberg G., Sagasser S., Sandberg R (2014). Full-length RNA-seq from single cells using Smart-seq2. Nat. Protoc..

[19] Clamer M., Tebaldi T., Lauria F., Bernabò P., Gómez-Biagi R.F., Marchioretto M (2018). Active ribosome profiling with ribolace. Cell Rep..

[20] Upton H.E., Ferguson L., Temoche-Diaz M.M., Liu X.M., Pimentel S.C., Ingolia N.T. (2021). Low-bias ncRNA libraries using ordered two-template relay: serial template jumping by a modified retroelement reverse transcriptase. Proc. Natl. Acad. Sci. U.S.A..

[21] Ferguson L., Upton H.E., Pimentel S.C., Mok A., Lareau L.F., Collins K. (2023). Streamlined and sensitive mono- and di-ribosome profiling in yeast and human cells. Nat. Methods.

[22] Mito M., Shichino Y., Iwasaki S (2023). Thor-ribo-seq: ribosome profiling tailored for low input with RNA-dependent RNA amplification. bioRxiv.

[23] Kaneko S., Miyoshi K., Tomuro K., Terauchi M., Tanaka R., Kondo S. (2024). Mettl1-dependent m^7^G tRNA modification is essential for maintaining spermatogenesis and fertility in *Drosophila melanogaster*. Nat. Commun..

[24] VanInsberghe M., Van den Berg J., Andersson-Rolf A., Clevers H., Van Oudenaarden A. (2021). Single-cell Ribo-seq reveals cell cycle-dependent translational pausing. Nature.

[25] Ozadam H., Tonn T., Han C.M., Segura A., Hoskins I., Rao S. (2023). Single-cell quantification of ribosome occupancy in early mouse development. Nature..

[26] Stuart T., Butler A., Hoffman P., Hafemeister C., Papalexi E., Mauck W.M. (2019). Comprehensive integration of single-cell data. Cell.

[27] Dingwall C., Lomonossoff G.P., Laskey R.A (1981). High sequence specificity of micrococcal nuclease. Nucleic Acids Res..

[28] Alagar Boopathy L.R., Jacob-Tomas S., Alecki C., Vera M (2022). Mechanisms tailoring the expression of heat shock proteins to proteostasis challenges. J. Biol. Chem..

[29] Shalgi R., Hurt J.A., Krykbaeva I., Taipale M., Lindquist S., Burge C.B (2013). Widespread regulation of translation by elongation pausing in heat shock. Mol. Cell.

[30] Iwasaki S., Floor S.N., Ingolia N.T (2016). Rocaglates convert DEAD-box protein eIF4A into a sequence-selective translational repressor. Nature..

[31] Shichino Y., Iwasaki S (2022). Compounds for selective translational inhibition. Curr. Opin. Chem. Biol..

[32] Jiang L., Schlesinger F., Davis C.A., Zhang Y., Li R., Salit M. (2011). Synthetic spike-in standards for RNA-seq experiments. Genome Res..

[33] Lovén J., Orlando D.A., Sigova A.A., Lin C.Y., Rahl P.B., Burge C.B (2012). Revisiting global gene expression analysis. Cell.

[34] Chen K., Hu Z., Xia Z., Zhao D., Li W., Tyler J.K (2015). The overlooked fact: fundamental need for spike-in control for virtually all genome-wide analyses. Mol. Cell. Biol..

[35] Risso D., Ngai J., Speed T.P., Dudoit S (2014). Statistical Analysis of Next Generation Sequencing Data.

[36] Han Y., David A., Liu B., Magadán J.G., Bennink J.R., Yewdell J.W. (2012). Monitoring cotranslational protein folding in mammalian cells at codon resolution. Proc. Natl. Acad. Sci. U.S.A..

[37] Andreev D.E., O’Connor P.B.F., Fahey C., Kenny E.M., Terenin I.M., Dmitriev S.E. (2015). Translation of 5’ leaders is pervasive in genes resistant to eIF2 repression. Elife.

[38] Popa A., Lebrigand K., Barbry P., Waldmann R (2016). Pateamine A-sensitive ribosome profiling reveals the scope of translation in mouse embryonic stem cells. BMC Genomics.

[39] Arpat A.B., Liechti A., De Matos M., Dreos R., Janich P., Gatfield D (2020). Transcriptome-wide sites of collided ribosomes reveal principles of translational pausing. Genome Res..

[40] Shieh A.W., Bansal S.K., Zuo Z., Wang S.H (2023). Transcriptome-wide profiling of acute stress induced changes in ribosome occupancy level using external standards. Plos One.

[41] Wang Y.J., Vaidyanathan P.P., Rojas-Duran M.F., Udeshi N.D., Bartoli K.M., Carr S.A. (2018). Lso2 is a conserved ribosome-bound protein required for translational recovery in yeast. Plos Biol..

[42] Wang Y.J., Gilbert W.V (2021). Quantitative comparisons of translation activity by ribosome profiling with internal standards. Methods Mol. Biol..

[43] Haneke K., Schott J., Lindner D., Hollensen A.K., Damgaard C.K., Mongis C. (2020). CDK1 couples proliferation with protein synthesis. J. Cell Biol..

[44] Hoerth K., Reitter S., Schott J (2022). Normalized ribo-seq for quantifying absolute global and specific changes in translation. Bio Protoc..

[45] Cattie D.J., Richardson C.E., Reddy K.C., Ness-Cohn E.M., Droste R., Thompson M.K. (2016). Mutations in nonessential eIF3k and eIF3l genes confer lifespan extension and enhanced resistance to ER stress in *Caenorhabditis elegans*. Plos Genet..

[46] Liu T.Y., Huang H.H., Wheeler D., Xu Y., Wells J.A., Song Y.S (2017). Time-resolved proteomics extends ribosome profiling-based measurements of protein synthesis dynamics. Cell Syst..

[47] Iwasaki S., Iwasaki W., Takahashi M., Sakamoto A., Watanabe C., Shichino Y (2019). The translation inhibitor rocaglamide targets a bimolecular cavity between eIF4A and polypurine RNA. Mol. Cell.

[48] Chen M., Asanuma M., Takahashi M., Shichino Y., Mito M., Fujiwara K (2021). Dual targeting of DDX3 and eIF4A by the translation inhibitor rocaglamide A. Cell Chem. Biol..

[49] Chhipi-Shrestha J.K., Schneider-Poetsch T., Suzuki T., Mito M., Khan K., Dohmae N (2022). Splicing modulators elicit global translational repression by condensate-prone proteins translated from introns. Cell Chem. Biol..

[50] Tomuro K., Mito M., Toh H., Kawamoto N., Miyake T., Chow S.Y.A. (2024). Calibrated ribosome profiling assesses the dynamics of ribosomal flux on transcripts. Nat. Commun..

[51] Smith J., Bartel D.P (2024). The G3BP stress-granule proteins reinforce the translation program of the integrated stress response. BioRxiv.

[52] Ingolia N.T., Lareau L.F., Weissman J.S (2011). Ribosome profiling of mouse embryonic stem cells reveals the complexity and dynamics of mammalian proteomes. Cell.

[53] Sears R.M., Nowling N.L., Yarbro J., Zhao N (2025). Expanding the tagging toolbox for visualizing translation live. Biochem. J..

[54] Tani H., Mizutani R., Salam K.A., Tano K., Ijiri K., Wakamatsu A. (2012). Genome-wide determination of RNA stability reveals hundreds of short-lived noncoding transcripts in mammals. Genome Res..

[55] Paulsen M.T., Veloso A., Prasad J., Bedi K., Ljungman E.A., Tsan Y.C. (2013). Coordinated regulation of synthesis and stability of RNA during the acute TNF-induced proinflammatory response. Proc. Natl. Acad. Sci. U.S.A..

[56] Ingolia N.T., Brar G.A., Rouskin S., McGeachy A.M., Weissman J.S (2012). The ribosome profiling strategy for monitoring translation *in vivo* by deep sequencing of ribosome-protected mRNA fragments. Nat. Protoc..

[57] Weinberg D.E., Shah P., Eichhorn S.W., Hussmann J.A., Plotkin J.B., Bartel D.P (2016). Improved ribosome-footprint and mRNA measurements provide insights into dynamics and regulation of yeast translation. Cell Rep..

[58] Zinshteyn B., Wangen J.R., Hua B., Green R (2020). Nuclease-mediated depletion biases in ribosome footprint profiling libraries. RNA.

[59] Alkan F., Silva J., Pintó Barberà E., Faller W.J (2021). Ribo-ODDR: oligo design pipeline for experiment-specific rRNA depletion in Ribo-seq. Bioinformatics.

[60] Choe D., Szubin R., Poudel S., Sastry A., Song Y., Lee Y. (2021). RiboRid: a low cost, advanced, and ultra-efficient method to remove ribosomal RNA for bacterial transcriptomics. Plos Genet..

[61] Gu H., Sun Y.H., Li X.Z (2021). Novel rRNA-depletion methods for total RNA sequencing and ribosome profiling developed for avian species. Poult. Sci..

[62] Chung B.Y., Hardcastle T.J., Jones J.D., Irigoyen N., Firth A.E., Baulcombe D.C. (2015). The use of duplex-specific nuclease in ribosome profiling and a user-friendly software package for Ribo-seq data analysis. RNA.

[63] Gu W., Crawford E.D., O’Donovan B.D., Wilson M.R., Chow E.D., Retallack H. (2016). Depletion of abundant sequences by hybridization (DASH): using Cas9 to remove unwanted high-abundance species in sequencing libraries and molecular counting applications. Genome Biol..

[64] Han P., Shichino Y., Schneider-Poetsch T., Mito M., Hashimoto S., Udagawa T (2020). Genome-wide survey of ribosome collision. Cell Rep..

[65] Wilkins O.G., Ule J (2021). Ribocutter: Cas9-mediated rRNA depletion from multiplexed Ribo-seq libraries. BioRxiv.

[66] Miettinen T.P., Björklund M (2015). Modified ribosome profiling reveals high abundance of ribosome protected mRNA fragments derived from 3’ untranslated regions. Nucleic Acids Res..

[67] Volbeda A., Lahm A., Sakiyama F., Suck D (1991). Crystal structure of penicillium citrinum P1 nuclease at 2.8 A resolution. EMBO J..

[68] Galmozzi C.V., Merker D., Friedrich U.A., Döring K., Kramer G (2019). Selective ribosome profiling to study interactions of translating ribosomes in yeast. Nat. Protoc..

[69] Wagner S., Bohlen J., Herrmannova A., Jelínek J., Preiss T., Valášek L.S. (2022). Selective footprinting of 40S and 80S ribosome subpopulations (Sel-TCP-seq) to study translation and its control. Nat. Protoc..

[70] Li S.H.J., Nofal M., Parsons L.R., Rabinowitz J.D., Gitai Z (2021). Monitoring mammalian mitochondrial translation with MitoRiboSeq. Nat. Protoc..

[71] Fujita T., Kurihara Y., Iwasaki S (2019). The plant translatome surveyed by ribosome profiling. Plant Cell Physiol..

[72] Schott J., Reitter S., Lindner D., Grosser J., Bruer M., Shenoy A. (2021). Nascent Ribo-Seq measures ribosomal loading time and reveals kinetic impact on ribosome density. Nat. Methods.

[73] Fremin B.J., Sberro H., Bhatt A.S (2020). MetaRibo-Seq measures translation in microbiomes. Nat. Commun..

